# Circadian Disruption: Potential Implications in Inflammatory and Metabolic Diseases Associated With Alcohol

**DOI:** 10.35946/arcr.v35.1.10

**Published:** 2013

**Authors:** Robin M. Voigt, Christopher B. Forsyth, Ali Keshavarzian

**Affiliations:** **Robin M. Voigt, Ph.D.,***is an assistant professor and Josephine M. Dyrenforth Chair of Gastroenterology; all at Rush University Medical Center, Chicago, Illinois.*; **Christopher B. Forsyth, Ph.D.,***is an assistant professor and Josephine M. Dyrenforth Chair of Gastroenterology; all at Rush University Medical Center, Chicago, Illinois.*; **Ali Keshavarzian, M.D.***is a professor and Josephine M. Dyrenforth Chair of Gastroenterology; all at Rush University Medical Center, Chicago, Illinois.*

**Keywords:** Alcohol consumption, alcohol-related disorders, disease factors, risk factors, circadian disruption, circadian rhythm, circadian clock, immune function, metabolism, inflammatory diseases, metabolic diseases, epigenetic mechanisms

## Abstract

Circadian rhythms are a prominent and critical feature of cells, tissues, organs, and behavior that help an organism function most efficiently and anticipate things such as food availability. Therefore, it is not surprising that disrupted circadian rhythmicity, a prominent feature of modern-day society, promotes the development and/or progression of a wide variety of diseases, including inflammatory, metabolic, and alcohol-associated disorders. This article will discuss the influence of interplay between alcohol consumption and circadian rhythmicity and how circadian rhythm disruption affects immune function and metabolism as well as potential epigenetic mechanisms that may be contributing to this phenomenon.

## Circadian Disruption and Society

The circadian clock is a sophisticated mechanism that functions to synchronize (i.e., entrain) endogenous systems with the 24-hour day in a wide variety of organisms, from simple organisms such as fungi up to the complex mammalian systems. Circadian rhythms control a variety of biological processes, including sleep/wake cycles, body temperature, hormone secretion, intestinal function, metabolic glucose homeostasis, and immune function. Functional consequences of modern-day society, such as late-night activity, work schedules that include long-term night shifts and those in which employees change or rotate shifts (i.e., shift work), and jet lag are substantial environmental disruptors of normal circadian rhythms. Fifteen percent of American workers perform shift work (Bureau of Labor Statistics 2005), indicating the pervasiveness of circadian disruption as a normal part of modern-day society. This change from the diurnal lifestyle of our ancestors to one that is more prominently nocturnal results in misalignment between natural rhythms based on the 24-hour day and behavioral activity patterns (i.e, circadian misalignment). Circadian misalignment has a significant detrimental effect on cell, tissue, and whole-organism function. These alterations can manifest in humans as chronic health conditions, such as metabolic syndrome,^1^ diabetes, cardiovascular disease, cancer, and intestinal disorders (Karlsson et al. 2001; Morikawa et al. 2005; Schernhammer et al. 2003; Penev et al. 1998; Caruso et al. 2004). The increased prevalence of diseases associated with circadian disruption underscores the need to better understand how circadian disruption can wreak havoc in so many different ways throughout the body.

## Central and Peripheral Circadian Rhythms

**Figure 1:**
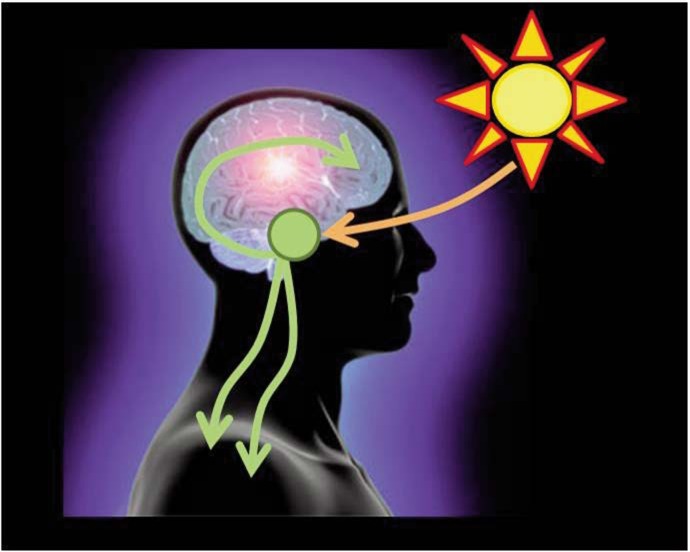
The suprachiasmatic nucleus (SCN) is the central circadian pacemaker. The SCN is located in the hypothalamus and is regulated by light signals from the eye. The SCN then affects a wide variety of physiological and behavioral outcomes.

**Figure 2:**
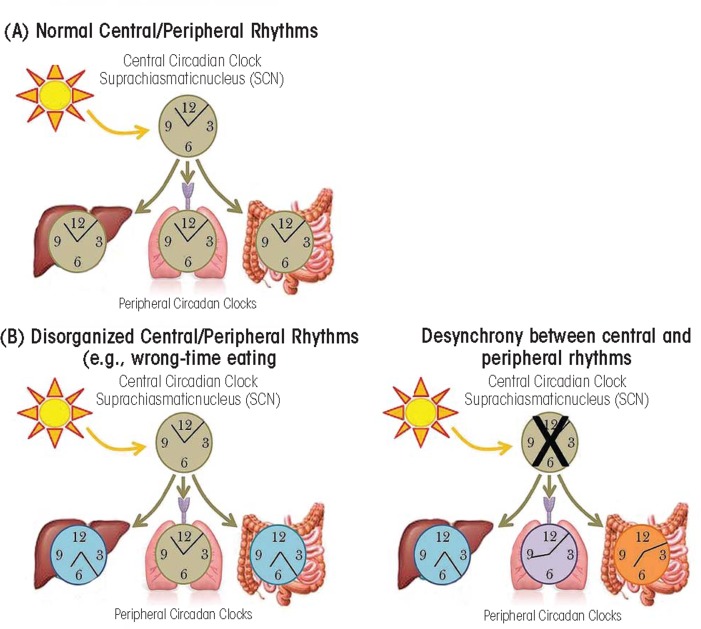
Central and peripheral circadian rhythms. **(A)** Under normal conditions, the central circadian clock in the suprachiasmatic nucleus which is entrained by light, then regulates peripheral circadian clocks. **(B)** Wrong-time eating can cause misalignment between the central circadian clock (entrained by light) and the peripheral circadian clocks entrained by food (illustrated here are intestine and liver). **(C)** When the central circadian clock is disrupted (e.g., due to lesion) peripheral circadian clocks will continue to cycle but will gradually become more misaligned with each other.

The master or central circadian clock (i.e., “pacemaker”) is located in the suprachiasmatic nucleus (SCN) in the anterior hypothalamus in the brain (Turek 1981) (see figure 1). The SCN is regulated by light stimulating retinal ganglion cells in the eye (Berson et al. 2002), and it is by this mechanism that light directs central circadian rhythms. Circadian rhythms are found in nearly every cell in the body, including the periphery, encompassing the immune system, heart, adipose tissue, pancreas, and liver (Allaman-Pillet et al. 2004; Boivin et al. 2003; Storch et al. 2002; Yoo et al. 2004; Zvonic et al. 2006). The SCN synchronizes circadian rhythms found in the periphery (figure 2A) via several mechanisms, including communication with nerve cells that influence visceral functions such as digestion, heart rate, etc., via direct release of the hormones oxytocin and vasopression into the general vasculature or indirectly via release of local signals that affect the release of hormones from the anterior pituitary gland (i.e., neuroendocrine and autonomic neurons) (Buijs et al. 2003). In addition, peripheral circadian rhythms can be regulated by external factors other than central light-entrained rhythms. For instance, abnormal feeding patterns can cause peripheral circadian rhythms (i.e., in the intestine and liver) to become misaligned with central rhythms if feeding is out of synch with the normal 24-hour pattern, a phenomenon that can be observed in both animals and humans (see figure 2B). Peripheral tissues express self-sustained rhythms that are able to function independent of the central clock in the SCN. For example, following SCN lesion that terminates central circadian rhythmicity, peripheral circadian clocks continue to demonstrate rhythmicity; however, peripheral rhythms become desynchronized from each other over time (Yoo et al. 2004) (see figure 2C). This internal misalignment is particularly detrimental because peripheral circadian clocks directly regulate up to 5 to 20 percent of the genome (i.e., so-called clock-controlled genes) (Bozek et al. 2009). Furthermore, reports indicate that 3 to 20 percent of the entire genome demonstrates 24-hour oscillations in gene expression, including genes critical for metabolic processes. This observation suggests that although not directly controlled by the circadian clock, genes are influenced as a consequence of rhythmic changes in transcription factors and transcriptional (i.e., the process of creating a complementary RNA copy of a sequence of DNA) and translational (i.e., when RNA is used to produce a specific protein) modifiers (i.e., proteins controlling the levels and activity of various processes including lipid metabolism and glucose synthesis) (Panda et al. 2002).

**Figure 3:**
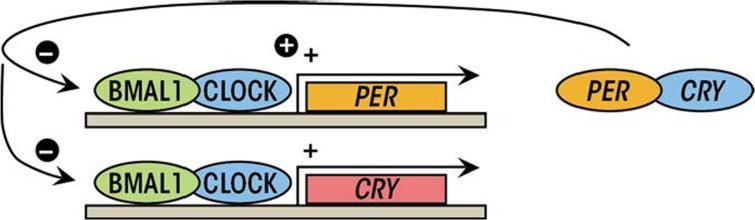
The molecular circadian clock. Transcription of the clock-controlled genes, including *Per* and *Cry* is initiated by the heterodimerization and binding of BMAL1 and CLOCK (the positive limb of the molecular circadian clock). Once sufficient amounts of PER and CRY have been produced, they dimerize and inhibit further BMAL1/CLOCK-mediated transcription (the negative limb of the molecular circadian clock).

At the cellular level, circadian rhythms originate from self-sustained, autoregulated, cyclic expressions of clock genes, which constitute the molecular clock. The molecular circadian clock consists of transcriptional activators and repressors—that is, proteins that stimulate and repress the production of proteins, respectively, in a cyclic process that is approximately 24-hours in duration (Reppert and Weaver 2002). The molecular circadian cycle is initiated when the transcriptional activators Clock and Bmal1 (Bunger et al. 2000) combine (i.e., heterodimerize) to stimulate the transcription of target circadian genes including period (*Per*) and cryptochrome (*Cry*) (i.e., *Per1* to *Per3* and *Cry1* and *Cry2*) as well as a host of other clock-controlled genes. When PER and CRY proteins accumulate in the cytosol, they heterodimerize and translocate to the nucleus where they act as transcriptional repressors to terminate CLOCK-BMAL1–mediated transcription, thus ending the molecular circadian cycle (van der Horst et al. 1999) (see figure 3). The cycle is further regulated by additional proteins, including the enzyme sirtuin 1 (SIRT1), a histone deacetylase that modifies circadian proteins or DNA by removing acetyl groups to alter gene expression. SIRT1 is sensitive to levels of the coenzyme nicotinomide adenine dinucleotide (NAD^+^), making NAD availability a potential regulator of the molecular circadian clock (Grimaldi et al. 2009). The details of this oscillating cycle are found elsewhere (Reppert and Weaver 2002).

Demonstrating the importance of the molecular circadian clock, mutations of the core circadian clock components can have a devastating effect on the function of the circadian clock. This is true for both *Bmal1* (Bunger et al. 2000) and *Clock* (Oishi et al. 2006). Likewise, molecular perturbation of the circadian clock (i.e., altering the *Clock, Bmal1, Per1, Per2, Cry1*, or *Cry2* expression via genetic manipulations including deleting or mutating the gene of interest to affect the levels of functional protein produced) disrupts normal circadian behavioral rhythms (Antoch et al. 1997; Bunger et al. 2000; van der Horst et al. 1999; Zheng et al. 2001). This article will discuss the influence of alcohol on circadian rhythms and how circadian-rhythm disruption affects immune function and metabolism, significant factors for alcohol-associated poor health outcomes. It also will discuss potential epigenetic mechanisms by which circadian disruption and alcohol may establish long-term changes in gene expression, resulting in adverse health outcomes.

## Alcohol and Circadian Rhythmicity

Circadian organization and stable circadian rhythms are vital for optimal health as numerous diseases are associated with circadian-rhythm disruption. Environmental factors such as shift work or jet lag are obvious disrupters of circadian rhythmicity. However, other environmental factors, such as alcohol consumption and the timing of food intake, can profoundly disrupt and disorganize circadian rhythmicity, which can be observed on behavioral, cellular, and molecular levels.

### Alcohol Disrupts Behavioral and Biological Circadian Rhythms

Alcohol has a dramatic effect on circadian rhythms. These circadian abnormalities include disrupted sleep/wake cycles in humans (Brower 2001; Imatoh et al. 1986) as well as disrupted circadian responses to light and abnormal activity patterns in rodents (Brager et al. 2010; Rosenwasser et al. 2005). The changes observed in behavioral patterns and responses to light may be the consequence of alcohol-induced disruption of normal tissue/organ function and neuroendocrine function. For example, normal cyclic patterns associated with body temperature (i.e., thermoregulation) (Crawshaw et al. 1998), blood pressure (Kawano et al. 2002), and characteristics of biochemical circadian rhythms including glucose and cholesterol rhythms (Rajakrishnan et al. 1999) are significantly affected by alcohol consumption. In addition, the circadian-driven production of hormones including melatonin (i.e., an endocrine hormone that is important in circadian entrainment) in rats (Peres et al. 2011) and humans (Conroy et al. 2012), corticosterone (i.e., a steroid hormone produced by the adrenal gland that responds to stress and regulates metabolism) (Kakihana and Moore 1976), and pro-opiomelanocortin (i.e., a polypeptide hormone that is a precursor to several hormones) (Chen et al. 2004) are disrupted by alcohol consumption. Alcohol-induced changes such as these have a profound impact on the functioning of a wide variety of peripheral organs and biological processes, which are dependent upon central circadian synchronization for proper function.

### Alcohol Disrupts the Molecular Circadian Clock

Not surprisingly, the changes observed in the behavioral and biological systems also are observed on the molecular level as a disrupted molecular circadian clock, an effect that is evident both in vitro and in vivo. Exposure of intestinal epithelial cells (i.e., Caco-2 cells, a widely used model of the human intestinal barrier) to alcohol increases the levels of circadian clock proteins CLOCK and PER2 (Swanson et al. 2011). Likewise, alcohol-fed mice have disrupted expression of *Per1–Per3* in the hypothalamus (Chen et al. 2004), human alcoholics demonstrate markedly lower expression of *Clock, BMAL1, Per1, Per2, Cry1*, and *Cry2* in peripheral blood mononuclear cells (i.e., immune cells) compared with nonalcoholics (Huang et al. 2010), and in humans alcohol consumption is inversely correlated to BMAL1 expression in peripheral blood cells (Ando et al. 2010). The alcohol-induced changes seem to have long-lasting effects on the circadian clock, particularly when the exposure occurs early in life, which may be the consequence of epigenetic modifications (discussed below). For example, neonatal alcohol exposure in rats disrupts normal circadian-clock expression levels and expression patterns over a 24-hour period (i.e., rhythmicity) (Chen et al. 2006; Farnell et al. 2008). These examples illustrate the ability of alcohol to have profound and long-lasting effects on clock-gene expression in multiple organs and tissues.

### Feed-Forward Cycle: Alcohol Promotes Circadian Disruption and Circadian Disruption Promotes Alcohol Consumption

Interestingly, circadian-clock disruption can promote alcohol consumption, which can further exacerbate this cycle. For example, *Per2* mutant mice exhibit increased alcohol consumption compared with wild-type counterparts (Spanagel et al. 2005), an effect attributed to altered reinforcement systems leading to enhanced motivation to consume alcohol. This may explain why humans with circadian disruption are more prone to substance abuse disorders (Trinkoff and Storr 1998). This phenomenon also sets up a potentially devastating cycle in which circadian disruption drives alcohol consumption, which further exacerbates circadian disruption.

## Mechanisms of Alcohol-Induced Circadian Disruption

The mechanisms by which alcohol disrupts circadian rhythmicity are likely a consequence of alcohol metabolism and alcohol-induced changes in intestinal barrier integrity.

### Consequences of Alcohol Metabolism

Alcohol is metabolized via several mechanisms, including the enzymes catalase, alcohol dehydrogenase (ADH), and cytochrome P450 (CYP2E1) (Lu and Cederbaum 2008). Although alcohol metabolism most prominently occurs in the liver, other tissues such as the stomach, intestine, and brain also play a role in this process. One consequence of alcohol metabolism that is particularly relevant for alcohol-induced disruption of circadian rhythmicity is a shift in the cellular NAD^+^/NADH ratio. SIRT1, which regulates the molecular circadian clock, is highly sensitive to the cellular NAD^+^/NADH ratio. Therefore, a perturbation in the availability of NAD^+^ (e.g., as a consequence of alcohol metabolism by ADH or as a consequence of aldehyde metabolism by acetaldehyde) would be one mechanism by which alcohol could disrupt the molecular circadian clock and resulting circadian rhythms.

### Alcohol, the Intestine, and Inflammation

Another mechanism by which alcohol can exert a negative influence on circadian rhythmicity is by promoting intestinal hyperpermeability. Alcohol disrupts intestinal barrier integrity in vitro (Swanson et al. 2011), in rodents (Keshavarzian et al. 2009), and humans (Keshavarzian et al. 1994, 1999). Intestinal hyperpermeability allows luminal bacterial contents such as endotoxin (e.g., lipopolysaccharide (LPS) to translocate through the intestinal epithelium into the systemic circulation. Endotoxin can disrupt circadian rhythms. LPS administered to rodents impairs the expression of *Per* in the heart, liver, SCN, and hypothalamus (Okada et al. 2008; Yamamura et al. 2010) and suppresses clock gene expression in human peripheral blood leukocytes (Haimovich et al. 2010). Thus, intestinal-derived LPS may be one mechanism by which alcohol disrupts circadian rhythmicity. In addition, LPS elicits a robust immune response in the periphery (Andreasen et al. 2008), and systemic inflammation disrupts normal circadian rhythmicity (Coogan and Wyse 2008). For example, tumor necrosis factor α (TNFα), a cytokine produced in response to endotoxins, disrupts normal locomotor behavior and sleep/wake cycles and alters expression of the molecular circadian clock in the liver (Cavadini et al. 2007). Thus, there are several plausible mechanisms by which alcohol-induced effects on the intestine may disrupt central and peripheral circadian rhythms.

It is clear that alcohol-induced effects on the intestine are highly detrimental to circadian rhythmicity. Interestingly, the reverse also is true in that the molecular circadian clock in the intestine influences alcohol-induced effects. Intestinal circadian rhythms are largely driven by feeding patterns (Hoogerwerf et al. 2007; Scheving 2000) and even the apical junctional complex (AJC) proteins, which regulate tight junctions (and thus intestinal permeability), are clock controlled in the kidney (Yamato et al. 2010). Alcohol exposure increases intestinal circadian gene expression, and knocking out *Clock* or *Per2* in intestinal epithelial cells (i.e., Caco-2 cells) prevents alcohol-induced intestinal hyperpermeability (Swanson et al. 2011). Taken together, alcohol—via metabolism products or intestine effects including endotoxemia and systemic inflammation—disrupts intestinal circadian rhythms, an effect that can further exacerbate internal misalignment.

## Circadian Rhythms and Immune Function

The immune system demonstrates robust circadian rhythmicity with daily variations in immune parameters, including lymphocyte proliferation, antigen presentation, and cytokine gene expression (Fortier et al. 2011; Levi et al. 1991). These rhythms seem to be sensitive to perturbations in circadian homeostasis, with differential effects depending on the cell type, model system, and outcome measure. For example, inhibition of *Per2* in natural killer (NK) cells (part of the innate immune system) decreases the expression of the immune effectors granzyme-B and porforin (i.e., critical cytotoxic components) (Arjona and Sarkar 2006*a*). Despite these changes, selective reduction of *Per2* in NK cells does not effect NK rhythmic production of the cytokine interferon-γ (IFNγ), which is important for the formation and release of reactive oxygen species. In contrast, whole-animal *Per2*-deficient mice have drastically disrupted IFNγ rhythms (Arjona and Sarkar 2006*b*). The IFNγ rhythmic disruption in *Per2*-deficient mice but not after selective reduction of *Per2* in isolated NK cells would be expected if IFNγ is dependent upon other circadian parameters, such as circadian fluctuations in hormones or temperature. Indeed, rhythmic hormones such as glucocorticoids and melatonin, which are significantly affected by circadian disruption, modulate immune function (Dimitrov et al. 2004; Srinivasan et al. 2005). *Per2*-deficient mice also demonstrate blunted LPS-induced septic shock compared with wild-type mice (Liu et al. 2006), indicating a functional change that has important biological implications. These studies demonstrate the significant disturbances that can occur as a consequence of a disrupted molecular circadian clock.

In addition to genetically manipulating circadian homeostasis, environmentally disrupting circadian rhythms also negatively affects immune function. For example, loss of regular sleep/wake cycles alters the normal circadian rhythmicity observed in immune cells (Bryant et al. 2004; Vgontzas et al. 2004) and increases the susceptibility to infections (Everson 1993; Mohren et al. 2002). Indeed, chronically shifting light/dark cycles in mice augments LPS-induced immune response, resulting in greater mortality compared with non–circadian-disrupted mice (Castanon-Cervantes et al. 2010).

Taken together, these studies provide evidence that circadian disruption can significantly, and typically negatively, influence immune function. Therefore, alcohol-induced circadian disruption may be a susceptibility factor for immune dysregulation, which may promote alcohol-associated inflammatory processes. Furthermore, the altered response to LPS has particular relevance in light of the alcohol-induced effects on intestinal permeability.

## Circadian Rhythms and Metabolic Syndrome

Although only a few metabolic genes are direct targets of circadian genes (Noshiro et al. 2007; Panda et al. 2002), the direct targets do include many transcription factors and other modulators of transcription and translation. These clock-controlled genes include factors regulating lipid and cholesterol biosynthesis, carbohydrate metabolism, oxidative phosphorylation, and glucose levels (Oishi et al. 2003; Panda et al. 2002).

Eating is an environmental factor that selectively affects peripheral circadian rhythmicity in the intestine and liver. Feeding at the incorrect time (e.g., late-night eating for humans) can result in internal circadian misalignment. For example, restricted feeding paradigms in which animals only have access to food during inappropriate times (i.e., during the light cycle for nocturnal rodents) results in misalignment between central light-entrained circadian rhythms (i.e., in the SCN) and peripheral food-entrained circadian rhythms, including those in the liver (Damiola et al. 2000). Recent studies suggest that this internal misalignment scenario is linked to weight gain, obesity, and metabolic syndrome. Indeed, mice fed during the inappropriate time gain more weight (Arble et al. 2009; Salgado-Delgado et al. 2010) than mice fed during appropriate time, despite similar activity levels and caloric intake (Arble et al. 2009). This phenomenon also is observed in humans; people who skip breakfast and have eating patterns shifted toward late-night eating tend to be more overweight than those who consume food during more appropriate time periods (Berkey et al. 2003; Ma et al. 2003).

Genetic abnormalities in the molecular circadian clock also are associated with metabolic disorders, including obesity, metabolic syndrome, and diabetes (Scott et al. 2008; Woon et al. 2007). For example, *Clock* mutant mice, which have disrupted circadian rhythms (Vitaterna et al. 1994), are obese and demonstrate characteristics of metabolic syndrome such as high cholesterol levels and high blood glucose (Turek et al. 2005). *Bmal1* mutant mice also have disrupted circadian rhythmicity (Bunger et al. 2000), disrupted adipogenesis (Shimba et al. 2005), and demonstrate markers of metabolic syndrome (e.g., higher levels of triglycerides and glucose) (Marcheva et al. 2010; Rudic et al. 2004). Similarly, mutations in *Cry* genes disrupt hormonal rhythms (Fu et al. 2005; Yang et al. 2009) and *Cry* mutants show markers of metabolic syndrome (Okano et al. 2009). It should be noted that although some of these mutant mice demonstrate disrupted locomotion and feeding behaviors (i.e., wrong-time feeding), the abnormalities seem to be attributable to mutations in the circadian clock machinery rather than to appropriate feeding times because mice (e.g., *Bmal1* mutant mice) that do exhibit normal activity/feeding patterns still exhibit markers of metabolic syndrome (Lamia et al. 2008; Marcheva et al. 2010).

In addition to these effects of circadian rhythms on indices of metabolism, it is also important to consider the effect of circadian disruption on the immune system because chronic inflammation is a prominent feature associated with metabolic syndrome. Thus, the immune dysfunction that occurs upon circadian rhythm disruption may be a predisposing or exacerbating factor for metabolic syndrome.

## Epigenetic Alterations: Circadian Rhythm Disruption and Alcohol

Epigenetics is the study of stable changes in gene expression that do not involve DNA sequence modifications but rather are the consequence of processes such as DNA methylation, histone modification (i.e., acetylation, methylation, phosphorylation, ubiquitinylation, ADP-ribosylation, and sumoylation), and noncoding micro-RNAs (miRNAs). These changes in gene expression are critical to optimize cellular function and for cellular development and differentiation. However, epigenetic changes also occur in response to environmental changes, including circadian rhythm disruption and alcohol use.

Shift work (i.e., chronic circadian disruption) is associated with an increased incidence of cancer. Potential mechanisms for this relationship include changes in melatonin levels and levels of circadian clock genes (Straif et al. 2007). However, epigenetics also may influence circadian rhythm disruption and thereby affect cellular function. Indeed, long-term shift work affects promoter methylation of the circadian genes *Clock* and *Cry2* (Zhu et al. 2011) with increased methylation of *Clock* (Hoffman et al. 2010*a*) and decreased methylation of *Cry* (Hoffman et al. 2010*b*) observed in cancer patients. Epigenetic changes also occur as a consequence of chronic circadian disruption in the promoter regions of genes encoding glucocorticoid receptors (important for hypothalamic–pituitary– adrenal axis function), TNFα(a cytokine critical for cell functioning and inflammation), and IFNγ (Bollati et al. 2010). Changes such as these may play a critical role in how chronic circadian disruption promotes cancer, inflammation, and metabolic disorders.

In addition to circadian-disruption–induced epigenetic changes, alcohol consumption is also associated with epigenetic modifications. Alcohol-induced DNA acetylation is observed in vitro in rat hepatocytes (Park et al. 2003), in vivo in rat hepatic stellate cells (Kim and Shukla 2005, 2006), lung, spleen, and testes (Kim and Shukla 2006). Similar to the increased cancer risk associated with chronic circadian disruption, alcohol-induced epigenetic changes are associated with the development of cancer. Indeed, colorectal cancer in high-alcohol– consuming humans is associated with high levels of promoter hypermethylation of several relevant genes when compared with low- or no-alcohol– consuming counterparts with colorectal cancer (van Engeland et al. 2003; Giovannucci et al. 1995). Similarly, alcohol-consuming individuals with head and neck cancer have hypermethylated gene promoters for specific genes of interest compared with non-alcohol–drinking individuals (Puri et al. 2005) and alcohol-dependent humans have hypermethylation of liver and peripheral blood cell DNA. Thus, it seems that both circadian disruption and alcohol consumption can affect long-term changes in gene expression via epigenetic modifications that may impact a wide variety of health outcomes.

## Summary and Future Directions

Circadian rhythms are a prominent and critical feature of cells, tissues, organs, and behavior that help an organism function most efficiently and anticipate things such as food availability. Therefore, it is not surprising that disrupted circadian rhythms or misalignment between central and peripheral circadian rhythms predispose and/or exacerbate a wide variety of diseases, including alcohol-associated disorders. One environmental factor that has been shown to have a disruptive effect on circadian rhythms is alcohol consumption. This disruption occurs at the molecular levels (i.e., changes in the expression levels of the circadian clock genes), also affects tissues and organs (e.g., changes in the cyclic pattern of hormones), and leads to overt behavioral changes. Thus, in the context of alcoholism, disrupted circadian rhythms may create a positive feedback loop that markedly exaggerates alcohol-induced immune/inflammatory-mediated diseases by (1) negatively influencing immune function and (2) promoting alcohol consumption that leads to further circadian-rhythm disruption. These changes are highly relevant because circadian-rhythm disruption has a substantial impact on immune function, which in turn has important implications for a wide variety of pathological conditions, including metabolic syndrome. A better understanding of how circadian rhythms influence such a wide variety of systems and bodily functions and how environmental factors such as alcohol use influence these processes is vital to our ever more circadian-disrupted society.

A better understanding of the mechanisms by which circadian disruption affects health outcomes such as cancer, inflammation, metabolic disease, and alcohol-induced pathology is critical. This information may lead to the development of chronotherapeutic approaches to prevent and/or treat a wide variety of conditions that are promoted or exacerbated by circadian-rhythm disruption and may lead to better risk stratification for individuals who are at risk for developing chronic conditions. Going forward, characterizing the epigenetic modifications that occur during chronic circadian disruption may be critical for understanding not only how disruption affects an individual but also how these modifications are passed on to offspring, which may influence the health of future generations. Thus, the issue of circadian disruption is vitally important for the health and well-being of current and future generations.

## Footnotes

1 Metabolic syndrom is a combination of disorders that can lead to diabetes and cardiovascular disease, characterized by abnormal levels of fat and/or cholesterol in the blood and insulin resistance.
